# Next-Generation Hydrogel Design: Computational Advances in Synthesis, Characterization, and Biomedical Applications

**DOI:** 10.3390/polym17101373

**Published:** 2025-05-16

**Authors:** Muhammad Mazhar Fareed, Sergey Shityakov

**Affiliations:** 1 Department of Computer Science, School of Science and Engineering, Università Degli Studi di Verona, 37134 Verona, Italy; m.mazharfareed97@gmail.com; 2 Laboratory of Bioinformatics, Department of Bioinformatics, Biocenter, Würzburg University, 97080 Würzburg, Germany

**Keywords:** hydrogels, in-silico design, computational modeling, molecular dynamics simulation, machine learning, bioinformatics, targeted therapeutic transport, engineered biological scaffolds, restorative healthcare technologies

## Abstract

Hydrogels are pivotal in advanced materials, driving innovations in medical fields, such as targeted drug delivery, regenerative medicine, and skin repair. This systematic review explores the transformative impact of in-silico design on hydrogel development, leveraging computational tools such as molecular dynamics, finite element modeling, and artificial intelligence to optimize synthesis, characterization, and performance. We analyze cutting-edge strategies for tailoring the physicochemical properties of hydrogels, including their mechanical strength, biocompatibility, and stimulus responsiveness, to meet the needs of next-generation biomedical applications. By integrating machine learning and computational modeling with experimental validation, this review highlights how in silico approaches accelerate material innovation, addressing challenges and outlining future directions for scalable, personalized hydrogel solutions in regenerative medicine and beyond.

## 1. Introduction

Hydrogels, characterized by their high-water content, biocompatibility, tunable mechanical properties, and ability to emulate the extracellular matrix, are pivotal in advancing biomedical applications, such as controlled drug delivery, tissue engineering, wound healing, and biosensing [1,2,3,4]. These versatile materials have garnered significant attention because of their ability to address complex clinical challenges, including targeted therapeutic delivery and regenerative medicine. However, traditional hydrogel development, which relies on empirical and iterative experimental approaches, is often resource intensive and time consuming, limiting the pace of innovation.

The advent of in silico methodologies has revolutionized hydrogel research by introducing computational tools that increase the precision, efficiency, and predictability of material design [5,6,7]. Techniques such as molecular dynamics simulations, finite element analysis, and machine learning enable researchers to model hydrogel behavior at the molecular and macroscopic scales, elucidating the structure-property-activity relationships critical for tailoring functionalities [8,9,10,11,12]. Molecular simulations provide insights into crosslinking mechanisms, network stability, mechanical properties, and degradation kinetics, whereas machine learning-driven models analyze extensive datasets to optimize polymer compositions for specific biomedical applications [13,14,15,16,17,18,19]. These computational strategies bridge theoretical predictions with experimental outcomes, offering a deeper understanding of hydrogel interactions with biological systems, including cells, tissues, and therapeutic agents, under physiological conditions [15,20,21]. This review systematically explores how in silico approaches are transforming hydrogel design, characterization, and application, paving the way for next-generation biomedical solutions.

## 2. Literature Parsing and Analysis

### 2.1. Focused Questions

This systematic review investigates whether in silico strategies, including molecular modeling and machine learning, significantly contribute to the rational drug design, simulation, and biomedical application of hydrogels. The focus is on their role in enhancing drug delivery efficiency, mechanical properties, and the biocompatibility of hydrogel systems used in various therapeutic contexts.

### 2.2. Eligibility Criteria

An extensive review of the literature was performed utilizing the PubMed, Google Scholar, Web of Science, and Scopus databases to retrieve relevant research. The search methodology incorporated controlled vocabulary terms and free-text keywords to maximize the identification of relevant publications.

The review included peer-reviewed articles and research articles written in English and published up to 2025. Emphasis has been placed on the literature from the past 20 years to reflect recent advancements in computational and data-driven techniques. Eligible studies had to meet specific inclusion criteria: (1) focus on hydrogel or hydrogel-like materials with explicit biomedical applications; (2) use computational modeling methods; (3) integrate machine learning techniques; and (4) present a clear methodological framework explaining the computational tools used and the results obtained or validated.

Studies were excluded if they lacked a computational or machine learning component, focused solely on nonhydrogel biomaterials, were purely experimental without in silico analysis, or were classified as letters to the editor, opinion pieces, or other non-peer-reviewed forms of publication.

## 3. Classification of Hydrogels

Hydrogels can be broadly divided into natural and synthetic categories (Table 1). Natural hydrogels derived from polysaccharides such as alginate and chitosan and proteins such as collagen and elastin are valued for their biocompatibility and biodegradability but often suffer from limitations in mechanical strength and batch-to-batch consistency [22,23,24]. Synthetic hydrogels, typically fabricated from polymers such as polyethylene glycol (PEG), polyvinyl alcohol (PVA), and polyacrylamide (PAM), offer enhanced mechanical and chemical stability but may have lower biocompatibility [25,26,27].

Hydrogels are also classified by their polymer architecture into homopolymeric, copolymeric, and interpenetrating polymer network (IPN) hydrogels, each with distinct advantages and limitations [28,29]. Their swelling behavior, which is crucial for drug delivery, is quantified by the equilibrium swelling ratio, often described by the Flory–Rehner model [30]. Hydrogels can be fabricated via physical or chemical crosslinking methods, and their degradation profiles can be tuned for biomedical applications [31,32,33,34].

Analytical techniques such as Fourier transform infrared (FTIR) spectroscopy, nuclear magnetic resonance (NMR) spectroscopy, X-ray diffraction (XRD), and ultraviolet–visible (UV-vis) spectroscopy are utilized to investigate the structural features and chemical composition of hydrogels [11,19,35,36]. The classification of hydrogels on the basis of physical and chemical parameters is presented in Table 2.

Hydrogels exhibit diverse physicochemical properties, including viscoelastic and mechanical characteristics, which directly influence their performance, stability, and bioactivity [19,35,36,38]. Techniques such as rheometry and dynamic mechanical analysis (DMA) are commonly used to assess the mechanical characteristics of hydrogels [39,40,41]. Morphological characterization methods, such as atomic force microscopy (AFM), transmission electron microscopy (TEM), light microscopy (LM), scanning electron microscopy (SEM), and micro-CT (microcomputed tomography), along with wide-angle X-ray scattering (WAXS) and small-angle X-ray scattering (SAXS), support the assessment of nanoscale morphology [11,42,43,44].

Polymer networks called hydrogels are essential for many biomedical applications (Table 3). They are primarily categorized into natural and synthetic types, each with distinct advantages and limitations. Natural hydrogels, including collagen, gelatin, hyaluronic acid (HA), and chitosan, are favored for their superior biocompatibility and biodegradability but often exhibit suboptimal mechanical properties [11,45]. Synthetic hydrogels, such as polyethylene glycol (PEG) derivatives, polycaprolactone (PCL), polyvinyl alcohol (PVA), and poly-N-isopropylacrylamide (PNIPAAm), offer increased mechanical durability but typically have poor biocompatibility [46,47].

### 3.1. Natural Hydrogels

Among natural hydrogels, collagen stands out because of its abundance in the extracellular matrix (ECM). Collagen-based hydrogels can be created by self-assembly triggered by temperature and pH, featuring cell-adhesion peptides, such as RGD (arginine-glycine-aspartic acid), which allow for integration with cellular receptors [48,49]. However, the limited mechanical qualities and batch-to-batch variability of collagen have prevented its wider use. Chemical cross-linking techniques, such as glycation and enzyme-mediated cross-linking, have been used to increase the stability and mechanical strength of collagen hydrogels [50].

Gelatin, derived from collagen through partial hydrolysis, shares many structural similarities with its parent protein but lacks the native triple helix. It is a preferred alternative to collagen because of its biodegradability and similarity in composition. Gelatin hydrogels can be chemically cross-linked through methods such as transglutaminase-induced cross-linking or photoinduced covalent bonding, particularly with methacrylate modifications [43,44,51].

Hyaluronic acid (HA), a highly hydrophilic ECM component, plays a significant role in cellular functions, including migration and differentiation, through its interaction with CD44 receptors. The molecular weight of HA is a key determinant of its biological activity. However, the natural inability of HA to undergo self-crosslinking limits its direct application in hydrogel formulations. To overcome this, HA polymers are often chemically modified, incorporating groups such as hydrazide, thiol, or methacrylate functionalities [52].

Alginate, a naturally occurring polymer obtained from brown seaweed, forms hydrogels when cross-linked with divalent cations, such as calcium. These alginate hydrogels exhibit a nanoporous structure that mimics the basement membrane, influencing cellular behaviors such as movement, differentiation, and dissemination [53].

### 3.2. Synthetic Hydrogels

Synthetic hydrogels are increasingly used in biological applications because of their promising qualities. PEG substitutes are frequently utilized for hydrogel production because of their affordability, ease of synthesis, and chemical modification potential. PEG hydrogels are typically formed through the photopolymerization of PEG diacrylate (PEGDA) but are inherently bioinert, necessitating chemical modifications to incorporate cell-binding motifs and biodegradable elements [54,55].

PVA is another widely used synthetic polymer for hydrogel formation because of its high biocompatibility and versatility in medical applications. However, PVA hydrogels suffer from low mechanical strength, limiting their effectiveness in certain applications. Methods such as double cross-linking and the addition of carbon nanotubes have been developed to improve the mechanical characteristics of PVA hydrogels [56].

PNIPAAm and other thermosensitive hydrogels undergo phase transitions in response to temperature changes, making them suitable for tissue engineering and drug delivery applications. While these hydrogels exhibit excellent tunability in response to temperature, their poor biodegradability and potential cytotoxicity hinder their widespread use in clinical settings [57].

Other synthetic hydrogels, such as PCL and polyurethane (PU), offer distinct advantages. PCL is known for its long degradation time and excellent biocompatibility, making it a preferred material for tissue scaffolds. PU hydrogels can be tailored by altering the chemical structure of polyols and isocyanates to achieve the desired mechanical properties [58,59].

Composite hydrogels, such as the chitosan-PEG-PNIPAAm blend, combine the advantages of multiple hydrogel materials, offering tunable properties, including increased mechanical strength and sensitivity to pH and temperature [60].

## 4. Hydrogel Physicochemical Properties and Their Impact on the Cell System

### 4.1. Stiffness

Stiffness is one of the most crucial properties of hydrogels in regulating cell behavior. The stiffness of the ECM varies significantly between tissues. For example, the brain typically has a stiffness of approximately 1 kPa, muscle tissue has a stiffness of approximately 10 kPa, and bone tissue can be as stiff as 100 kPa. In contrast, diseased tissues, such as tumor tissues, often show altered stiffness, which has been associated with disease progression and metastasis. Hydrogels can be engineered to mimic the stiffness of specific tissues, thus providing a supportive environment for cells. The stiffness of a hydrogel scaffold can influence various aspects of cell behavior, including morphology, proliferation, migration, and differentiation [61].

The ability of cells to sense substrate stiffness is mediated primarily by integrins, focal adhesion complexes, and the cytoskeleton. Cells modulate their adhesion and internal contractility in response to changes in substrate stiffness, ensuring mechanical homeostasis. Mechanotransduction signaling pathways that are influenced by stress include integrin-mediated FAK signaling, the RhoA/ROCK pathway, the YAP/TAZ pathway, and the Wnt/beta-catenin signaling pathway, all of which play vital roles in regulating cell fate [62].

**Figure 1:**
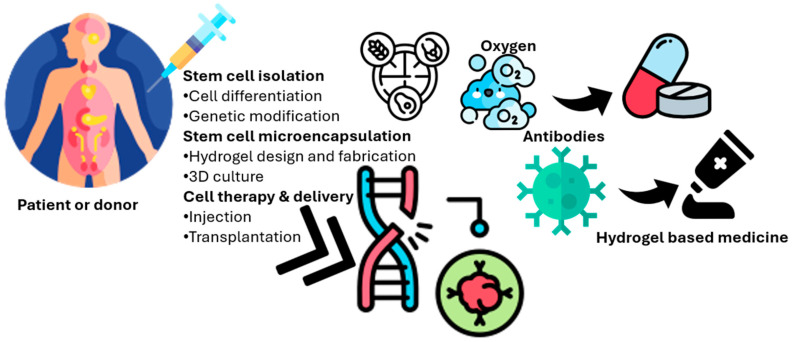
Schematic representation of stem cell microencapsulation within a biocompatible hydrogel matrix for cell therapy. The process involves stem cell isolation, encapsulation, nutrient and oxygen diffusion, immune protection, and controlled delivery of therapeutic cells via injection or transplantation.

Additionally, stem cell microencapsulation for cell therapy involves the process of stem cell microencapsulation within a hydrogel matrix for therapeutic applications. The process begins with stem cell isolation, potentially involving cell differentiation or genetic modification, from a patient or donor (Figure 1). These cells are then encapsulated within a biocompatible hydrogel, allowing for nutrient and oxygen diffusion while protecting the cells from the host’s immune response (e.g., antibodies and immune cells). Growth factors can be incorporated into hydrogels to promote cell survival and function. Waste products are able to diffuse out of the hydrogel. Finally, this hydrogel-based system can be delivered via injection or transplantation for cell therapy, offering a controlled and protective environment for the cells to exert their therapeutic effects. The molecular structure exemplifies a hydrogel component. This microencapsulation strategy enables controlled release and localized delivery, enhancing the efficacy and safety of stem cell-based therapies [16,39].

### 4.2. Stimulus-Responsive Hydrogels

Stimulus-responsive hydrogels, often called “smart” hydrogels, have notable benefits for regulating cell life in response to modifications in the environment. These hydrogels can undergo physical or chemical changes upon exposure to stimuli such as temperature, pH, light, or ionic strength. This responsiveness enables them to modify pore size, alter scaffold stiffness, or release bioactive chemicals under control, all of which can have an immediate impact on cellular behavior. Smart hydrogels have shown promise in several applications, such as tissue regeneration, wound healing, and controlled drug delivery [63].

While significant progress has been made in understanding the role of the physicochemical properties of hydrogels in regulating cell behavior, several challenges remain. It is still difficult to produce hydrogels that accurately replicate the intricacy of natural ECMs. Furthermore, the influence of hydrogel properties on cell behavior can vary depending on the cell type, environmental factors, and dynamic nature of in vivo conditions. Future research should focus on the development of more sophisticated hydrogel systems that integrate multiple cues (mechanical, chemical, and biological) to more effectively control cellular responses and tissue development [64].

## 5. Trends in Hydrogel Customization for Regenerative Medicine

The strategic research of bioactive substances, such as growth factors, bioligands, and peptides, to modify specific cellular behaviors has been highlighted by recent studies [65]. This approach enables hydrogels to actively promote biological processes, such as osteogenesis, angiogenesis, and neural differentiation [66]. The effect of bioactive molecule concentration on cell differentiation rates can be captured via logistic growth models, which consider factors such as the maximal differentiation rate, sensitivity coefficient, and threshold concentration.

Innovative fabrication techniques such as 3D bioprinting, advanced crosslinking methods, and rapid prototyping have facilitated the production of hydrogel scaffolds with finely tuned geometries and functionalities [67]. The mechanical and geometric properties can be computationally predicted via finite element modeling (FEM), ensuring compatibility with diverse tissue engineering applications, including vascular, cardiac, spinal, and urethral regeneration.

**Figure 2:**
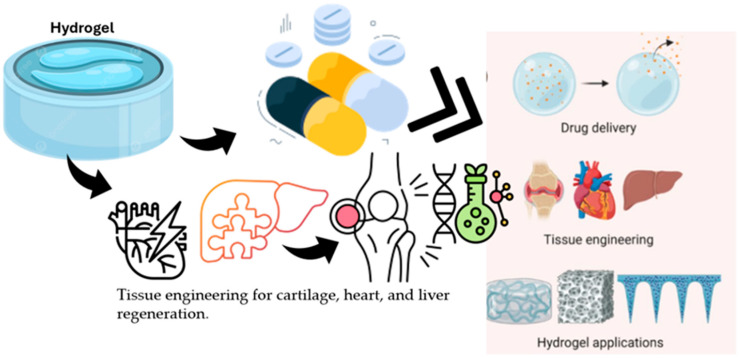
Illustration of a swollen hydrogel network showing its water absorption capacity and drug encapsulation mechanism. The diagram highlights the structural features enabling controlled drug release and applications in tissue engineering for cartilage, heart, and liver regeneration.

Hybrid hydrogel systems that integrate both organic and inorganic components along with nanomaterials offer mechanical resilience and controlled biofunctionality. These complex formulations are often described mathematically through multivariable optimization functions that balance mechanical strength, electrical conductivity, and cell compatibility [68]. The structure of hydrogels, their drug encapsulation and release mechanisms, and their biomedical applications underscore their importance in the biomedical field. The swollen hydrogel network is particularly notable for its capacity for water absorption and potential for drug incorporation (Figure 2). Additionally, drug encapsulation within the hydrogel matrix facilitates controlled drug release and supports tissue engineering in various formats, including cartilage, heart, and liver applications [69].

In addition, biological conditions are gaining significant prominence. The gelation dynamics of these materials can be modeled via time-dependent functions that incorporate the equilibrium gel modulus and the gelation rate constant. Porosity and oxygenation in scaffold design are critical parameters that significantly influence cell viability and integration with host tissues. These relationships are governed by diffusion principles, in which the partial pressure of oxygen and the availability of nutrients depend on pore size and scaffold thickness [70].

In hepatic tissue engineering, controlling the hydrogel microarchitecture and mechanical properties optimizes hepatic function. Biocomposite gelatin films with growth factors support epithelial regeneration [71]. Electrospun nanofiber scaffolds mimic ECM structures, aiding tissue regeneration suitable for computational modeling [39,72].

Carrageenan-based hydrogels assist in wound repair, whereas decellularized heart ECM preserves angiogenic proteins for cardiac regeneration [73]. Patterned gelatin-methacrylate and polyethylene glycol diacrylate hydrogels form epidermal models, and 3D microchanneled gelatin hydrogels promote cardiomyocyte functionality [50,74].

Hydrogel carriers from human hair-derived keratins deliver growth factors for muscle loss treatment. Chitosan hydrogels with stem cells enhance cardiac regeneration, and antioxidant DNA hydrogels deliver interleukin-33 for diabetic wound healing [75,76].

Hydrogels absorb and retain large amounts of water, making them useful in medical and industrial applications. Their behavior depends on their composition and structural response. Chitosan-based hydrogels are thermosensitive, alginate hydrogels are photosensitive, and PEG-based hydrogels respond to reactive oxygen species. DNA-based hydrogels are biosensitive, and peptide-based hydrogels respond to pH, temperature, and ion concentration [77,78].

Hydrogels can be categorized by structure and composition: homopolymers, copolymers, or multipolymers, and amorphous, semicrystalline, or crystalline (Table 4). Crosslinking can be chemical or physiological, affecting mechanical behavior and substance movement. Their forms vary, such as those of soft matrices or thin films, and their electrical properties can be nonionic, ionic, or mixed [50].

Polyvinyl alcohol (PVA) hydrogels are notable for their stability, strength, and safety. They are used in tissue engineering, drug delivery, wound dressings, wearable sensors, personal care, biodegradable packaging, energy storage, and water treatment [79].

Heparin hydrogel microwells optimize hepatocyte culture, and injectable hyaluronic acid-based hydrogels support spinal cord repair. Interpenetrating networks of hyaluronic acid and fibrin improve scaffold properties and cellular proliferation [80], demonstrating the potential of ECM-based scaffolds for tissue regeneration.

## 6. Dynamic Mechanical Properties in Tissue Development

Morphological behavior, including differentiation, is strongly influenced by the mechanical characteristics of tissues and polymers. Native tissues exhibit dynamic mechanical properties that change over time, impacting cellular responses. During chick heart development, mesodermal tissue stiffens as it matures, with its elastic modulus increasing from 0.9 kPa to 8.2 kPa between thirty-six and 408 h postfertilization [81].

To more closely resemble natural tissue conditions and direct cellular behavior appropriately, hydrogels can recreate changes in tissue stiffness via a variety of techniques.

### 6.1. Incorporating Dynamic Properties into Hydrogels

One approach to incorporating dynamic mechanical properties into hydrogels is through the use of thiol-modified hyaluronic acid (HA) hydrogels, which can be engineered to mimic the stiffening behavior of tissues such as the chick heart. Research by Young and Engler demonstrated that hydrogels with dynamic properties upregulated the expression of mature cardiac markers in cells, especially compared with static polyacrylamide hydrogels [49]. Other methods to introduce dynamic properties into hydrogels involve the use of supramolecular chemistry and self-assembly techniques, where specific peptides are incorporated into the hydrogel to enable responses to various physical, chemical, and biological stimuli.

### 6.2. Supramolecular Chemistry in Hydrogel Design

Supramolecular chemistry offers a promising strategy for designing hydrogels that can respond dynamically to various stimuli, such as light, temperature, pH, or enzyme cleavage. For example, research by Stupp and colleagues demonstrated that supramolecular nanofibers could effectively deliver bone-regenerating growth factors, such as BMP-2, while maintaining dynamic responsiveness [82]. Additionally, peptide sequences inside these materials can imitate complicated biological molecules, such as vascular endothelial growth factor (VEGF), which is critical for blood vessel creation [83]. Another notable study revealed that high-density presentation of a laminin-derived epitope on nanofibers selectively differentiated neural progenitors into neurons [84]. These findings highlight the potential of incorporating supramolecular materials into hydrogels to create dynamic systems that adapt to their surroundings, offering controlled delivery of biological molecules and promoting tissue regeneration.

Researchers are using 3D biomimetic printing to investigate dynamic biomaterials in addition to supramolecular substances, a cutting-edge technique that adds time to traditional 3D printing. For example, cellulose fibrils have been used to print hydrogels with anisotropic swelling behavior, allowing them to change shape over time upon immersion in water. Using this technique, 3D shapes inspired by nature, such as the New Guinean native Dendrobium helix orchid, have been produced [85]. Although 3D biomimetic printing systems have not yet been widely applied in regenerative medicine, their potential is vast. The ability to print dynamic structures that react to environmental conditions could revolutionize the field.

### 6.3. Future Applications of Regenerative Medicine in 3D Bioprinting

**Figure 3:**
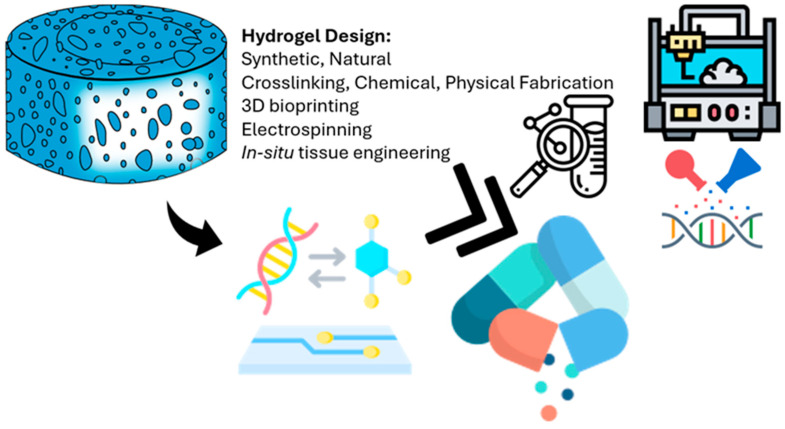
Overview of how hydrogel scaffolds are designed and fabricated for use in clinical applications. The hydrogel shown at the top left represents the basic structure, which can be made from either synthetic or natural materials. Key factors in designing these scaffolds include the type of crosslinking (chemical or physical) and the fabrication methods used, such as 3D bioprinting (top right), electrospinning, and other microengineering techniques. The arrow pointing to the left shows how bioactive molecules and genetic material can be added to the hydrogel to improve its biological function. The double arrow highlights how hydrogel systems are increasingly being integrated with lab-on-a-chip platforms and other precise engineering tools to create tissue-specific designs. Finally, the icon at the bottom right represents the ultimate goal: applying these engineered hydrogels directly in patients for uses like tissue regeneration and targeted drug delivery.

The future potential of 3D biomimetic systems in regenerative medicine is promising, especially for clinical applications such as heart valve repair and maxillofacial surgery. For example, significant efforts have been made to develop tissue-engineered heart valves to replace damaged or stenotic valves due to congenital conditions or disease, although these therapies often require invasive open-heart surgeries for implantation. However, a 3D bioprinted heart valve could be inserted via a minimally invasive procedure, forming its functional shape in situ and potentially revolutionizing heart valve repair by reducing the need for complex surgical interventions. Additionally, orbital floor fractures, which can lead to complications such as enophthalmos if not properly treated, can be repaired during maxillofacial procedures via 3D bioprinted structures that match a patient’s unique anatomy, allowing for minimally invasive placement that later expands to assume the full shape of the tissue [86]. The development of therapeutic drugs for human use remains challenging, with only 10.4% of all drugs examined in phase I clinical trials subsequently approved for use [87]. Over the past two decades, tissue engineering (TE)-based therapies have emerged as promising strategies for functional tissue replacement, with 371 clinical trials involving hydrogels registered globally by mid-2017, 69 of which focused on TE applications [88]. While these efforts have shown limited success in simpler organ structures such as the skin, cornea, urethra, bladder, and blood vessels, developing advanced TE strategies for more complex tissues faces challenges such as ensuring adequate oxygen and nutrient supply through vascularization, combining different cell types in precise spatial arrangements, obtaining unique mechanical characteristics, and integrating engineered tissues with host tissue. Hydrogel-based scaffolds show great promise in addressing these challenges [89,90]. Furthermore, designing hydrogels for clinical applications involves selecting appropriate materials, whether synthetic or natural, determining suitable crosslinking methods (chemical or physical) to achieve the desired mechanical strength and stability, and utilizing fabrication techniques such as 3D bioprinting, electrospinning, and in situ tissue engineering to create hydrogels with specific architectures (Figure 3). The ultimate goal is to transform these engineered hydrogels into effective clinical systems, underscoring the interdisciplinary nature of hydrogel research [91].

### 6.4. Cartilage Repair

**Figure 4:**
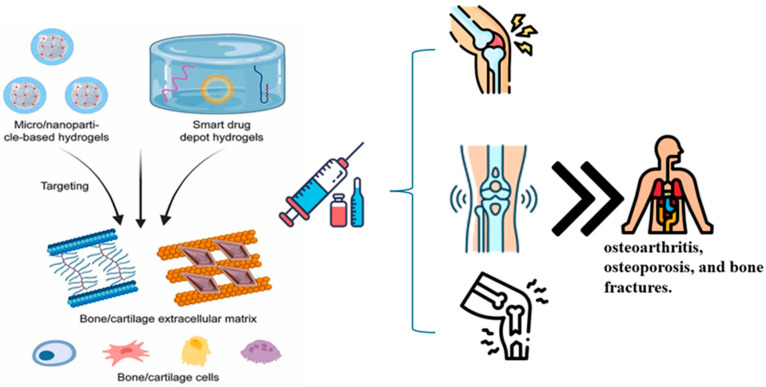
Schematic representation of hydrogel-based therapies for bone and cartilage regeneration. The illustration highlights two therapeutic strategies: (1) micro/nanoparticle-based hydrogels and (2) smart drug depot hydrogels. These systems are designed for targeted delivery to the bone and cartilage extracellular matrix, enhancing local interaction with resident cells (e.g., chondrocytes, osteoblasts). Additionally, systemically delivered formulations support broader therapeutic effects, including targeting bone marrow. These approaches aim to treat degenerative and traumatic musculoskeletal conditions such as osteoarthritis, osteoporosis, and bone fractures.

Repairing specific cartilage is one of the most promising uses of hydrogels in clinical TE. There is a significant need for efficient cartilage regeneration techniques because osteoarthritis affects 13.8% of people over 60 years of age worldwide [92]. Articular cartilage is an ideal candidate for TE approaches because it is relatively simple in structure, avascular, aneural, and composed of a single cell type. Numerous studies have explored the use of hydrogels to regenerate cartilage. These materials include thermoresponsive composites such as chitosan-PVA and hydrogels made from natural ECM constituents such as type I collagen. Other synthetic hydrogel systems, such as a PLGA–PEG–PLGA-based hydrogel, have also shown promise [93]. In one notable clinical trial, Sharma et al. developed a photoreactive, PEGDA-based hydrogel [77], which was injected into focal cartilage defects in the medial femoral condyle of 15 patients. This approach was combined with microfracture surgery, which enabled autologous cells to infiltrate the hydrogel. Compared with microfracture alone, the results demonstrated improved tissue regeneration, with greater tissue filling and better organization. Patients also reported reduced pain, a critical factor for clinical success. However, long-term follow-up is needed to confirm whether this treatment can prevent the progression of osteoarthritis [94]. Preclinical trials using various hydrogel systems have also yielded encouraging results. In rabbit models, for example, cartilage healing was facilitated by a synthetic PLGA–PEG–PLGA hydrogel loaded with kartogenin and mesenchymal stem cells (MSCs). Similarly, an oligo(poly(ethylene-glycol) fumarate)-based hydrogel containing MSCs [95] facilitated more hyaline-like cartilage regeneration than scaffolds alone in porcine models [96]. Additionally, hydrogel-based therapies for bone and cartilage regeneration involve the use of hydrogels to treat bone- and cartilage-related diseases (Figure 4). Two primary types of hydrogels, micro/nanoparticle-based hydrogels and smart drug depot hydrogels, can specifically target the bone/cartilage extracellular matrix and cells. These hydrogels are effective in treating common bone conditions such as osteoarthritis, osteoporosis, and bone fractures, highlighting the potential of local hydrogel injection as a therapeutic approach. Furthermore, the delivery of drugs via hydrogels to the bone marrow has been demonstrated, indicating a systemic treatment strategy [96].

## 7. Integration of Classical Molecular Modeling Methods in Hydrogel Research

Classical molecular modeling has transformed next-generation hydrogel design by enabling precise digital prototyping, multiscale characterization, and optimization of synthesis processes for biomedical applications. Techniques such as molecular dynamics (MD), density functional theory (DFT), finite element analysis (FEA), and computational fluid dynamics (CFD) provide insights into hydrogel behavior at the atomic, molecular, and macroscopic levels, accelerating innovations in drug delivery, tissue engineering, and biosensing [40,69,97,98,99,100].

MD simulations elucidate the nanoscale interactions, modeling of polymer crosslinking, pore structure, and water absorption dynamics critical for hydrogel swelling and mechanical strength. For example, MD distinguishes bound, intermediate, and free water states, informing stimulus-responsive hydrogel design, although challenges remain in simulating micron-sized pores owing to computational limitations [101,102,103]. Accurate MD relies on molecular force fields (e.g., AMBER, CHARMM, CVFF, PCFF), with integration algorithms such as Verlet, velocity Verlet, or Leap-Frog balancing accuracy and efficiency [80,104,105,106]. DFT, a quantum mechanical approach, probes electronic structures and water diffusion and enhances the design of hydrogels with tailored electronic or hygroscopic properties [107,108]. FEA discretizes hydrogel systems to predict mechanical responses, swelling behavior, and stress distribution, enabling the design of scaffolds with tissue-specific stiffness [109]. CFD optimizes manufacturing processes by simulating fluid and heat transport during hydrogel synthesis [110].

Multiphysics modeling integrates mechanical, thermal, and fluidic interactions, simulating complex hydrogel behaviors under physiological conditions. This approach supports the development of next-generation hydrogels with tunable degradation kinetics and stimuli responsiveness [111]. Response surface methodology (RSM) further refines synthesis conditions, optimizing parameters such as the polymer concentration and crosslinking density for enhanced performance [112,113]. By replacing labor-intensive trial-and-error methods, these computational tools enable rapid prototyping and characterization, paving the way for scalable, high-performance hydrogels in regenerative medicine and precision therapeutics.

## 8. Integration of Artificial Intelligence in Hydrogel Research

Artificial intelligence (AI) in computational science enables systems to perform complex tasks, such as data analysis, decision-making, and predictive modeling, which traditionally require human expertise. By leveraging sophisticated algorithms and expansive datasets, AI facilitates data-driven insights critical for next-generation hydrogel design [90,97,114,115]. Since the mid-20th century, AI has progressed through distinct phases: the symbolic era prioritized logic-based systems, the knowledge-based era focused on rule-driven expertise, and the statistical learning era introduced machine learning (ML) techniques, including random forests, support vector machines (SVMs), and deep learning. Reinforcement learning has recently gained prominence, significantly impacting materials science [116].

ML, a core AI discipline, encompasses supervised, unsupervised, and reinforcement learning, employing models such as neural networks, SVMs, and transformers [26,117,118]. In hydrogel research, AI-driven predictive models correlate composition, structure, and performance, optimizing synthesis and characterization for biomedical applications [91,119]. Techniques such as gradient descent and evolutionary algorithms identify optimal formulations that enhance hydrogel stability and functionality [92].

AI-powered hydrogels advance drug delivery, biosensing, bioprinting, wound healing, and tissue engineering by improving safety and accelerating material discovery [93]. Unlike traditional labor-intensive methods, AI enables high-throughput modeling, leveraging the ECM-mimicking properties of hydrogels, such as high water content, biocompatibility, and tunable mechanics [19,35,94,95,96]. Diverse polymer integrations further refine the mechanical and biological characteristics of the material [4,101].

Mathematical models, which incorporate variables such as the polymer concentration, molecular weight, and temperature, quantify hydrogel property relationships. Nanoparticle incorporation enhances mechanical and biological performance, whereas advanced fabrication supports dynamic scaffold design [51]. AI optimizes scaffold engineering through polymer chemistry, nanotechnology, and computational bioengineering [66,67].

For example, PEG-based hydrogels promote tissue regeneration, whereas natural polymers excel in terms of biocompatibility and ECM emulation [110,120,121]. Moreover, gelatin methacryloyl (GelMA) hydrogels offer tunable mechanics, influencing matrix metalloproteinase expression, and alginate-based systems support cryopreservation and regeneration [101,102,122,123,124]. Composite and hybrid hydrogels incorporating nanoparticles enhance mechanical and electrical properties, increasing bioactivity [13,14,125,126].

Supervised ML methods, including artificial neural networks (ANNs), deep neural networks (DNNs), convolutional neural networks (CNNs), and extreme gradient boosting (XGBoost), model input-output relationships and identify key variables [5,10,26,53,122,123,127,128,129,130,131]. Unsupervised methods such as principal component analysis (PCA) reduce data dimensionality, revealing patterns [132,133]. Reinforcement learning optimizes design parameters, whereas support vector regression (SVR) and least squares SVM (LS-SVM) refine property predictions [5,10,125,134,135,136,137]. Ensemble models, such as random forests (RFs) and SVMs, ensure robust analysis, minimizing overfitting [5,50,54,111,136,138,139,140]. Hybrid approaches integrating ANNs, fuzzy logic, and rule-based systems enhance optimization, predicting self-assembly and biofunctionality [5,121,125,134,137,141,142,143,144,145,146,147]. Collectively, these AI and ML advancements enable precise, scalable, and innovative hydrogel designs, revolutionizing biomedical applications by accelerating material discovery and optimizing performance for personalized therapeutic solutions.

## 9. Conclusions and Future Outlook

This systematic review explores the transformative role of in silico hydrogel design, emphasizing the integration of computational and AI techniques in advancing hydrogel properties for biomedical applications. AI, ML, and high-throughput computational screening have accelerated the development of materials for tissue regeneration, wound healing, drug delivery, and biosensing. These advancements enable accurate predictions of hydrogel characteristics, reducing the reliance on traditional experimental methods. AI-driven models and optimization strategies enhance the design of hydrogels with improved properties. Computational tools such as molecular dynamics simulations and finite element analysis contribute to a comprehensive understanding of hydrogels, supporting the development of next-generation systems. Customizing hydrogel properties for specific therapeutic purposes improves clinical outcomes and expedites material innovation.

The future of in-silico hydrogel design holds promise with evolving AI models enhancing predictive capabilities and material discovery. Integrating patient-specific data into hydrogel design methods promotes personalized medicine. Hybrid hydrogels combining nanomaterials and bioactive chemicals will unlock new possibilities in drug delivery and tissue engineering. AI-assisted design techniques enable precise control at the microscale, bolstering applications in organ regeneration and biosensing. Computational modeling and AI tools promise to revolutionize biomedical and healthcare applications, stimulating innovation across industrial sectors. Hydrogels have great potential in clinical applications, but overcoming scientific, regulatory, and practical challenges is essential.

Advanced numerical simulations have improved our understanding of hydrogel properties, complementing traditional methods and fostering innovation. AI and ML have emerged as powerful tools in hydrogel development, enabling data-driven design and optimization. Cutting-edge AI techniques such as deep learning open new possibilities for tailored hydrogels. However, challenges remain in ensuring data quality and reliability, bridging the gap between simulations and experimental validation, and addressing ethical aspects of AI and ML in hydrogel design. Collaboration across disciplines is crucial to developing standardized datasets and shared benchmarks, driving innovation, and improving patient outcomes.

## Figures and Tables

**Table 1:** Classification of hydrogels on the basis of various criteria [27].

Category	Subcategories
Source	Natural polymers and synthetic polymers
Polymer composition	Interpenetrating network, homopolymeric, copolymeric
Crosslinking	Physical, chemical
Degradability	Biodegradable, nonbiodegradable
Structure	Amorphous, semicrystalline
Physical Aspects	Film, gel, matrix, micro/nanoparticles
Stimulus responsiveness	Physical (such as temperature), chemical (such as pH), biochemical (e.g., antigen)
Charge	Cationic, anionic, and neutral

**Table 2:** Classification of hydrogels on the basis of physical and chemical parameters [37].

Category	Subcategories
Polymer composition	Homopolymeric (e.g., poly(N-isopropylacrylamide)), copolymeric (e.g., poly(PEGMA-monomethyl), heteropolymeric (e.g., poly(vinyl alcohol)-gelatin), hybrid (different polymers or phases)
Network structure	Physical cross-linking, chemical cross-linking
Stimulus responsiveness	Physical stimuli (temperature), chemical stimuli (pH, ionic strength), and biochemical stimuli (anti-gen, enzyme)
Physical aspects	Micro/nanoparticles: microbeads, nanogels, film: electrospun mats, matrix, scaffolds, and gel: injectable drug-loaded hydrogels
Configuration of chains	Noncrystalline (random structure in amorphous regions), semicrystalline (combination of amorphous and ordered/crystalline regions)

**Table 3:** Summary of typical hydrogel materials and their physicochemical properties [47].

Hydrogel Type	Material	Properties	Applications
Natural	Collagen	Biocompatible, biodegrad- able, low mechanical strength	Tissue engineering and wound healing
Natural	Gelatin	Biodegradable, poor me-chanical strength, cross-linked forms available	Tissue scaffolds for re generative medicine
Natural	Hyaluronic Acid	Hydrophilic, cell-binding sites, varying molecular weights influence function	Drug delivery and tissue repair
Natural	Alginate	Bioinert, tunable me-chanical properties, cross-linkable with divalent cations	Cell encapsulation and tissue scaffolds
Synthetic	PEG derivatives	Easy to synthesize, bioin-ert, modifiable, tunable mechanical properties	Drug delivery and tissue engineering
Synthetic	PVA	Biocompatible, low me-chanical strength, enhanced by cross-linking	Contact lenses and artificial joints
Synthetic	PNIPAAm	Temperature-sensitive, poor biodegradability, cytotoxicity issues	Drug delivery and cell carriers

**Table 4:** The classification of hydrogels is based on their source, structure, chemical nature, and stimulus-responsiveness [44,50,68].

Category	Subcategories
Source of monomer/polymer	Natural, synthetic, and hybrid (nanocomposite)
Configuration	Amorphous (noncrystalline), semicrystalline, and crystalline
Polymeric composition	Homopolymeric, copolymeric, and multipolymer
Type of cross-linking	Chemical (covalent bonding) and physical (noncovalent bonding)
Pore size between polymer systems	Nonporous, microporous, and superporous
Stimuli responsiveness	Thermosensitive (chitosan), photosensitive (alginate), glucose-responsive (agarose), pH-responsive (hyaluronic acid), enzyme-responsive (PVA), ROS-responsive (PEG), biosensitive (DNA-based), multifunctional-responsive (peptide-based)
